# A feedback regulatory model for RifQ-mediated repression of rifamycin export in *Amycolatopsis mediterranei*

**DOI:** 10.1186/s12934-018-0863-5

**Published:** 2018-01-29

**Authors:** Chao Lei, Jingzhi Wang, Yuanyuan Liu, Xinqiang Liu, Guoping Zhao, Jin Wang

**Affiliations:** 1 CAS Key Laboratory of Synthetic Biology, Institute of Plant Physiology and Ecology, Shanghai Institutes for Biological Sciences, Chinese Academy of Sciences, 500 Caobao Road, Shanghai, 200233 China; 2 Shanghai Tolo Biotechnology Company Limited, Shanghai, 200233 China; 3 Department of Microbiology and Li Ka Shing Institute of Health Sciences, The Chinese University of Hong Kong, Prince of Wales Hospital, Shatin, New Territories, Hong Kong SAR China

**Keywords:** Rifamycin, *Amycolatopsis mediterranei*, *rifQ*, Export, Feedback regulation

## Abstract

**Background:**

Due to the important role of rifamycin in curing tuberculosis infection, the study on rifamycin has never been stopped. Although RifZ, which locates within the rifamycin biosynthetic cluster, has recently been characterized as a pathway-specific regulator for rifamycin biosynthesis, little is known about the regulation of rifamycin export.

**Results:**

In this work, we proved that the expression of the rifamycin efflux pump (RifP) was regulated by RifQ, a TetR-family transcriptional regulator. Deletion of *rifQ* had little impact on bacterial growth, but resulted in improved rifamycin production, which was consistent with the reverse transcription PCR results that RifQ negatively regulated *rifP*’s transcription. With electrophoretic mobility shift assay and DNase I Footprinting assay, RifQ was found to directly bind to the promoter region of *rifP*, and a typical inverted repeat was identified within the RifQ-protected sequences. The transcription initiation site of *rifP* was further characterized and found to be upstream of the RifQ binding sites, well explaining the RifQ-mediated repression of *rifP*’s transcription in vivo. Moreover, rifamycin B (the end product of rifamycin biosynthesis) remarkably decreased the DNA binding affinity of RifQ, which led to derepression of rifamycin export, reducing the intracellular concentration of rifamycin B as well as its toxicity against the host.

**Conclusions:**

Here, we proved that the export of rifamycin B was repressed by RifQ in *Amycolatopsis mediterranei*, and the RifQ-mediated repression could be specifically relieved by rifamycin B, the end product of rifamycin biosynthesis, based on which a feedback model was proposed for regulation of rifamycin export. With the findings here, one could improve the antibiotic yield by simply inactivating the negative regulator of the antibiotic transporter.

**Electronic supplementary material:**

The online version of this article (10.1186/s12934-018-0863-5) contains supplementary material, which is available to authorized users.

## Background

Actinomycetes produce a wide range of secondary metabolites, including more than 70% of known antibiotics [1]. It is generally thought that antibiotics are produced by the hosts to inhibit competing microorganisms in the environment, guaranteeing competitive growth advantages [2]. However, the antibiotics could also be toxic to the host cells, and therefore the synthesis of antibiotics is usually under strict control [3]. Besides, the hosts have developed several other strategies to protect themselves, which may include chemical modification of the antibiotics, mutation of the intracellular target of the antibiotics and export of the antibiotics [4, 5]. In many cases, two or more strategies are employed to provide the best protection of the host from the threat of the antibiotic toxicity.

Rifamycin belongs to ansamycin, consisting of a naphthalene chromophore bridged by a long aliphatic chain [6], and it specifically binds to the β-subunit of bacterial RNA polymerase (RNAP) with high affinity, blocking the mRNA synthesis [7, 8]. As rifamycin and its derivatives are especially effective against mycobacteria, they are still used as the first-line drugs for treating infections caused by tuberculosis, leprosy and the mycobacterium avium complex (MAC) [9, 10]. Rifamycins are mainly produced by *Amycolatopsis mediterranei* [11], although there was a report that a marine bacterium, *Salinispora arenicola*, also possessed the ability to synthesize rifamycins [12]. To facilitate the study of the rifamycin biosynthesis, the nearly 100-kb rifamycin biosynthetic cluster (*rif*) was first sequenced in *A. mediterranei* S699 [13]. Totally, 44 genes were revealed in the cluster, including five large polyketide synthase genes, eight genes associated with the 3-amino-5-hydroxybenzoic acid (AHBA) biosynthesis, two regulators and several tailor genes [13–15]. Recently, RifZ, one of the two regulators within *rif* cluster, was found to be involved in rifamycin biosynthesis and was characterized as a pathway-specific regulator for *rif* transcription [16]. However, it is still unclear whether the export of rifamycin is regulated. Besides, the biological function of RifQ, the other regulator annotated within *rif* cluster, is also unknown.

*rifQ* locates downstream of *rifP*, which encodes a rifamycin efflux pump [17], and the two genes co-transcribe [16]. Unlike RifZ, which is a LuxR-family regulator, RifQ belongs to the TetR family. In this study, we systematically studied RifQ and characterized it as a specific repressor for *rifP*’s transcription, demonstrating its regulatory role in rifamycin export. Similar to what found with TetR [18], the end product of rifamycin biosynthesis, rifamycin B, was found to specifically decrease the RifQ binding affinity against the promoter of *rifP*, which therefore derepressed the rifamycin export to reduce the intracellular concentration of rifamycin B as well as the antibiotic toxicity.

## Results

### Disruption of *rifQ* dramatically improved the rifamycin production

**Fig. 1:**
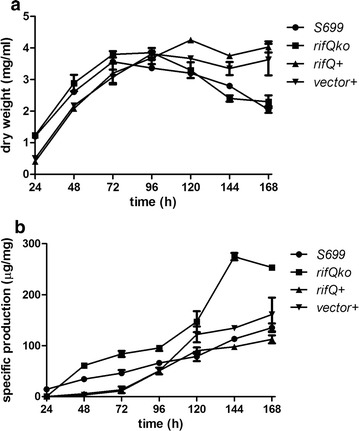
The growth and rifamycin B yield of different *A. mediterranei* strains. **a** Measurement of the growth curves. Bacterial dry weight was calculated every 24 h. **b** Measurement of the specific production of rifamycin B at different time points. Erythromycin was added into the culture medium of *rifQ*+ and *vector*+. S699, the wild type; *ΔrifQ*, the *rifQ* null mutant; *rifQ*+, *ΔrifQ* transformed with pDXM4-*rifQ*; *vector*+, *ΔrifQ* transformed with the blank vector. Three biological replicates were performed for each strain

The RifQ regulator belonged to the TetR-family and was highly homologous to the VarR in *Streptomyces virginiae* (63% identity, 78% similarity) (Additional file 1: Figure S1) [19]. To investigate the biological function of RifQ, we in-frame knocked out *rifQ*, and measured both the growth and rifamycin production of the *rifQ* null mutant (*ΔrifQ*). Compared with the wild type, *ΔrifQ* showed no significant difference in growth rate (Fig. 1a), but produced more than twofold rifamycin B (Fig. 1b). We also noticed the rifamycin production was partially restored in *rifQ*+, and this incomplete complementation of the phenotype was probably caused by the unstable expression of *rifQ*, which was cloned on an episomal plasmid pDXM4. For example, the transcription of *rifQ* was relatively stable throughout the tested growth stages (i.e., 24, 48 and 72 h) in the wild type, but decreased dramatically in *rifQ*+ (Additional file 2: Figure S2).

We also attempted to determine the intracellular rifamycin concentration in both the wild type and *ΔrifQ*, but failed. Consistently, in a previous report [17], where *rifP* was knocked down and the export of rifamycin was decreased, the authors also failed to detect intracellular rifamycin.

### RifQ regulated the rifamycin export through direct repression of *rifP*’s transcription

**Fig. 2:**
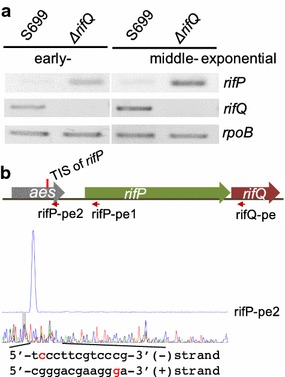
Transcriptional analysis of *rifP.* **a** RT-PCR analysis of *rifP* in both S699 and *ΔrifQ*. *rpoB* was used as an internal control. **b** Characterization of the *rifP* transcriptional initiation site (TIS) using the primer extension assay. Primers designed for both *rifP* and *rifQ* were illustrated in the schematic chart, and the *rifP* TIS was indicated with a red vertical line in the chart and shown in red in the sequence. The primer extension result using rifP-pe2 was shown, and results with rifP-pe1 could be found in Additional file 6: Figure S6

As the first characterized TetR-family regulator (TFR), TetR regulates the expression of the tetracycline transporter TetA [18, 20]. As RifQ was also a TFR, we wondered whether it could regulate the expression of the rifamycin transporter, which was encoded by *rifP* [17], the upstream gene of *rifQ*. Reverse transcription (RT)-PCR assay was then performed to analyze the transcriptional level of *rifP* in both the wild type and *ΔrifQ*. At two different growth phases (i.e., the early- and middle-exponential phases), the transcription of *rifP* was significantly increased in *ΔrifQ* in comparison with the wild type (Fig. 2a), demonstrating RifQ as a repressor for *rifP*.

The transcription of *rifP* and *rifQ* was previously found to initiate from *rif35*, forming a large *rif35* operon transcribing from *rif35* to *rifQ* in *rif* cluster (Additional file 3: Figure S3) [16]. However, except *rifP*, the transcription of other genes in this large operon was not significantly repressed by RifQ (Additional file 4: Figure S4), which indicated that *rifP* had its own promoter. To check this possibility, primer extension assay was employed to determine the possible transcription initiation site (TIS) of *rifP*. Two primers, which were complementary to different regions in *rifP* promoter, were designed for the experiments, and consistent results were obtained (Fig. 2b and Additional file 5: Figure S5). *rifP* was proved to transcribe from the − 447th nucleotides relative to the *rifP* coding sequence (CDS). Besides, a primer was also designed for *rifQ*, but no TIS was detected (data not shown), indicating the co-transcription of *rifP* and *rifQ*.

**Fig. 3:**
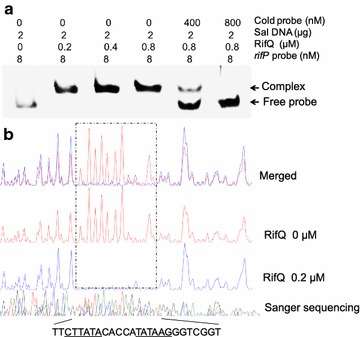
RifQ specifically bound to the *rifP* promoter region. **a** EMSA analysis of RifQ binding of *rifP* promoter. From lane 1 to lane 4, the *rifP* probe was incubated with different concentrations of RifQ protein, i.e., 0, 0.2, 0.4 and 0.8 µM, respectively. In lanes 5 and 6, 50- and 100-folds of cold *rifP* probe was added into the reaction system for specific competition, respectively. **b** Characterization of the RifQ-protected DNA sequences through DNase I Footprinting assay. The *rifP* probe was incubated with (blue line) or without (red line) RifQ protein, and was then partially digested by DNase I. The RifQ-protected sequences were shown and the proposed RifQ binding sites were underlined. To prevent non-specific binding between RifQ and the DNA probes, sheared salmon sperm DNA was added in above reactions

To further investigate the molecular mechanism of RifQ-mediated repression of *rifP*’s transcription, both electrophoretic mobility shift assay (EMSA) and DNase I Footprinting assays were employed, using purified recombinant RifQ. As revealed by the gel filtration analysis, RifQ functioned as a homodimer (i.e. 46.9 KD) (Additional file 6: Figure S6), which was like most other TFRs [21]. Based on the EMSA results, RifQ was found to specifically bind to the *rifP* promoter, and all *rifP* probe was shifted when only 0.2 µM RifQ was added in the reaction mix (Fig. 3a). Then, the RifQ-protected DNA sequences (5′-ACCGACCCTTATATGGTGTATAAGAA-3′) were precisely determined by DNase I Footprinting assay, extending from the − 61st to − 35th relative to the *rifP* CDS. An inverted repeat DNA binding motif, which was commonly known for TFR binding [22], was identified and underlined in the RifQ-protected sequences (Fig. 3b). As the *rifP* TIS located upstream of the RifQ binding sites, RifQ must act as a roadblock to block the transcriptional elongation of *rifP* (Additional file 7: Figure S7).

### Rifamycin B but not rifamycin SV weakened the DNA binding affinity of RifQ

**Fig. 4:**
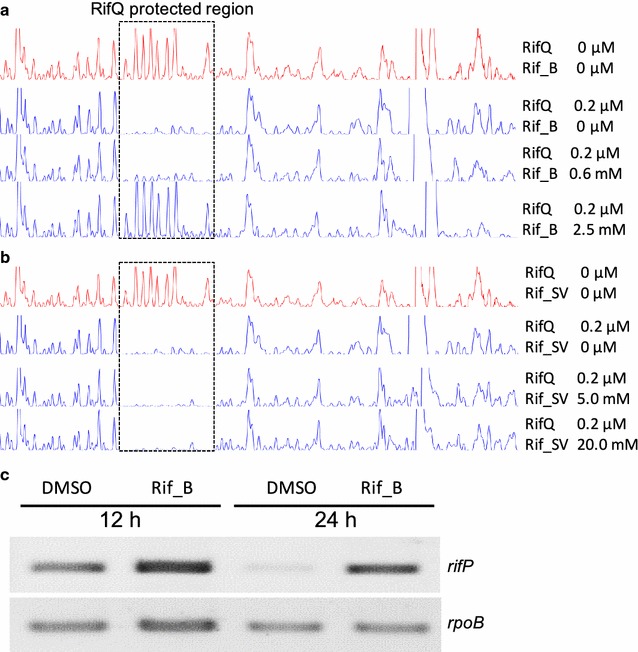
Rif_B not Rif_SV remarkably weakened the DNA binding affinity of RifQ. The RifQ binding affinity to the promoter regions of both *rifP* was tested with the addition of either Rif_B (**a**) or Rif_SV (**b**). Probes were incubated with (blue line) or without (red line) RifQ protein, and were then partially digested by DNase I. The RifQ-protected regions were indicated by dashed boxes. Rif_B but not Rif_SV appeared to weaken the RifQ binding affinity. As Rif_B and Rif_SV were dissolved in DMSO, DMSO was added in the blank group. To prevent non-specific binding between RifQ and DNA probes, sheared salmon sperm DNA was added in above reactions. **c** Rif_B inactivated the regulatory activity of RifQ in vivo. RT-PCR assay was employed to analyze the transcriptional levels of *rifP* in *LYZL11* (Additional file 9: Table S1), a *rifA* mutant strain, which did not produce rifamycins. Either DMSO or Rif_B was added into the growth medium and samples were collected at both 12 and 24 h after rifamycin addition. As indicated in the DNase I Footprinting assay, 2.5 mM Rif_B was able to completely deactivate the RifQ binding activity, and therefore a final concentration of 2.5 mM Rif_B was added into the growth medium

As TFRs are usually regulated by their corresponding allosteric effectors [23], we tested the effect of rifamycin B (Rif_B), the final product of *rif* cluster, on the RifQ binding affinity to its target promoter. RifQ was pre-incubated with Rif_B, and the RifQ binding affinity was determined through DNase I Footprinting assay. When the final concentration of Rif_B reached 0.6 mM, the RifQ binding affinity to *rifP* promoter slightly decreased, and RifQ completely lost the DNA binding affinity when Rif_B concentration increased to 2.5 mM (Fig. 4a). However, rifamycin SV (Rif_SV), a clinically important intermediate product of rifamycin biosynthesis, was unable to reduce the RifQ binding affinity to *rifP* promoter even when up to 20 mM Rif_SV was added (Fig. 4b). Therefore, the DNA binding activity of RifQ was specifically regulated by Rif_B but not its analogue Rif_SV.

Then, we further tested the role of Rif_B in inactivating RifQ in vivo. Rif_B was directly added into the culture medium of *LYZL11*, which was a *rifA* frameshift mutant and produced no rifamycin [24]. As expected, the *rifP*’s transcription increased at both 12 and 24 h upon the addition of Rif_B (Fig. 4c), which was similar to those found in *ΔrifQ* (Fig. 2a) and therefore demonstrated that Rif_B could effectively inactivate RifQ in vivo. Meanwhile, the effect of Rif_SV on *rifQ* transcription was also tested in vivo; however, no difference could be found between conditions with and without Rif_SV supplementation (Additional file 8: Figure S8), which was consistent with the results of the DNase I Footprinting assay.

## Discussion

In our research, RifQ was proved to negatively regulate the transcription of *rifP*, and *rifP*’s TIS was determined to decipher the molecular mechanism of RifQ-mediated repression.

**Fig. 5:**
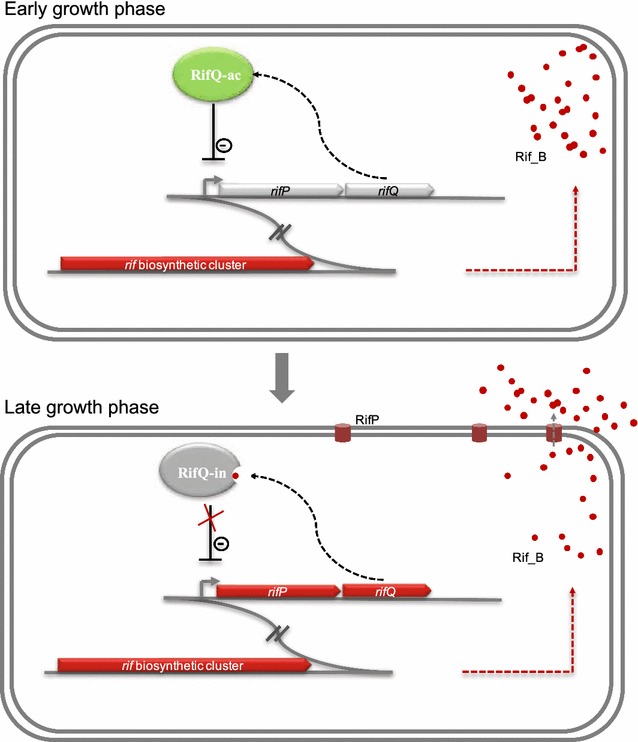
A proposed feedback model for RifQ-mediated regulation of rifamycin biosynthesis and export in *A. mediterranei*. At the early growth phase, RifQ represses Rif_B export, accelerating the accumulation of intracellular Rif_B. When the intracellular Rif_B concentration reaches a threshold, rifamycin B was able to inactivate RifQ by either direct binding or an unknown manner, and the export of Rif_B is remarkably promoted, reducing the intracellular concentration of Rif_B

Based on these findings, a feedback model is proposed for the regulation of rifamycin export (Fig. 5). At the early growth stage, the intracellular Rif_B concentration is low, and the RifQ activity is unaffected by Rif_B, resulting in active RifQ (RifQ-ac). In such a circumstance, RifQ-ac negatively regulates *rifP* transcription, repressing rifamycin export, which leads to the increased Rif_B accumulation within the cells. When cells enter the late growth phase, the intracellular Rif_B concentration reaches a threshold and inactivates RifQ by either direct binding or an unknown manner. Then, the inactivated RifQ (RifQ-in) releases from the promoter region of *rifP*, which derepressed *rifP* transcription to accelerate Rif_B export. Therefore, with this self-regulatory feedback model, the concentration of intracellular Rif_B is controlled under a relatively low level, protecting hosts from the toxicity of endogenous rifamycin.

In fact, the feedback autoregulation is widely used by the producers to regulate the antibiotic biosynthesis, avoiding the accumulation of intracellular antibiotic. For example, antibiotics such as jadomycin B and daunorubicin regulate their own biosynthesis, where the end products can inactivate the activators to slow down the antibiotic biosynthesis [25–27].

Moreover, deletion of RifQ resulted in a more than twofold improvement of rifamycin production in medium because of the overexpression of *rifP*. In *ΔrifQ*, the accelerated export of rifamycin may reduce the intracellular rifamycin concentration and relieve other possible feedback inhibition of rifamycin biosynthesis, which could possibly be raised by the accumulation of intracellular rifamycin. As a result, the rifamycin biosynthesis may keep sustained in *ΔrifQ*, leading to increased final production. Therefore, one may use similar strategies to improve the industrial production of other antibiotics whose export is specifically regulated by a repressor.

## Conclusions

Export of antibiotics is an important means for the producer to reduce the intracellular antibiotic concentration as well as its toxicity. Although both scientific research and industrial production of rifamycins have lasted for decades, little is known about the regulation of the export of this important antimycobacterial antibiotic. Here in this study, RifQ was proved to directly bind to the *rifP* promoter to repress its transcription. As RifP was a rifamycin pump protein, RifQ therefore repressed the export of rifamycin. Besides, we also found the RifQ-mediated regulation to *rifP* could be relieved by Rif_B, indicating Rif_B as an allosteric compound to induce the conformational change of RifQ. Based on the findings above, we concluded a feed-back model to illustrate the regulation of the rifamycin B export in *A. mediterranei* S699.

## Methods

### Bacterial strains, media, and primers

*Amycolatopsis mediterranei* S699 and its mutants were grown in Bennet medium [28] at 30 °C. When needed, apramycin (50 µg/mL), erythromycin (200 µg/mL), kanamycin (50 µg/mL) and ampicillin (100 µg/mL) were added. All strains and plasmids used in this study were listed in Additional file 9: Table S1, and primers were listed in Additional file 10: Table S2.

### RNA extraction and RT-PCR

*Amycolatopsis mediterranei* strains were grown in liquid Bennet medium for 48 h at 30 °C, and then cells were inoculated into a fresh liquid Bennet medium with the final OD_600_ diluted to 0.2. To culture *vector*+ and *rifQ*+ strains, erythromycin was added into the medium at a final concentration of 200 µg/mL to maintain the plasmids. Cells were harvested at different time points, representing the early- and middle-exponential growth phases, respectively. Total RNA was then extracted with the ZR Fungal/Bacterial RNA MiniPrep (Zymo Research), and further treated with RNase-free DNase I (TaKaRa) to remove trace genomic DNA. Random hexamers were employed for RT, and 3 µg total RNA was reverse-transcribed into cDNA in a 30-µL reaction system, using the Super-Script III reverse transcriptase (Thermo Fisher Scientific). PCR was performed using 20 ng cDNA as the template in a 20-µL reaction system, using either oneTaq DNA polymerase (NEB) or EasyTaq DNA polymerase (Transgen), and *rpoB* was employed as an internal control. The gene transcriptional level was monitored by agarose gel electrophoresis, followed by ethidium bromide staining. A negative control was performed following the same procedures except the addition of reverse transcriptase was omitted.

### Knockout and complementation of *rifQ* in *A. mediterranei* S699

About 2-kb fragments of both the upstream and downstream regions of *rifQ* were amplified from S699 genome using two pairs of primers of rifQkoP1/rifQkoP2 and rifQkoP5/rifQkoP6, respectively, and the apramycin resistance cassette was amplified from plasmid pBCAm (ColE1 ori, chloramphenicol and apramycin resistance), using primers of rifQkoP3/rifQkoP4. The above three fragments were then mixed together with the *EcoR*V-linearized pBluescript SK II in equal moles, which were subsequently assembled with the Ezmax seamless assembly kit (Tolo Biotech.), generating the plasmid p*rifQko* for *rifQ* knockout. The plasmid was first denatured by heating before being electroporated into *A. mediterranei* S699 competent cells, and transformants were selected on plates supplemented with apramycin. Colonies were verified by PCR amplification with primers of rifQkoCK1 and rifQkoCK2, employing the wild type as a control.

To construct pDXM4-*rifQ* plasmid for complementation of *rifQ*, the *rifP* promoter region and the *rifQ* coding sequence were individually amplified from S699 genome, using primers of rifQkoCP1/rifQkoCP2 and rifQkoCP3/rifQkoCP4, respectively. Amplicons were then seamlessly assembled with the *Eco*RV-linearized pDXM4 [29] to obtain plasmid pDXM4-*rifQ*. The pDXM4-*rifQ* was transformed into the *rifQ* null mutant by electroporation, obtaining the *rifQ*+ strain. Plasmid pDXM4 was also transformed into *ΔrifQ*, and the transformant was employed as a negative control.

### Measurement of bacterial growth curves and rifamycin yield

*Amycolatopsis mediterranei* strains were first grown in liquid Bennet medium for 48 h at 30 °C, and then inoculated into a fresh liquid Bennet medium with the final OD_600_ diluted to 0.2. The growth of strains was measured every 24 h, and the growth curve was generated on the basis of the dry weight.

The measurement of rifamycin production was similar to the methods developed by Pasqualucci et al. [30]. Briefly, 100 µL cell cultures were centrifuged at 12,000 rpm for 10 min, and 10 µL supernatant was added with 90 µL vitamin C solution or NaNO_2_ solution, respectively. The mixtures were placed at room temperature for 10 min before being measured at A425 using the Varioskan Flash (Thermo Fisher Scientific) and the NaNO_2_ group was taken as a blank control. The yield of rifamycin was calculated based on a standard curve obtained with pure rifamycin B. The measurement was carried out every 24 h, at the same time point for measuring bacterial growth curve.

Intracellular rifamycin was extracted according to the method described by Bhat et al. [31], and its concentration was determined by both the same method described above and the HPLC method, using the Agilent 1200 system equipped with the proshell EC-C18 column (4.6 × 50 mm, 2.7 µm). A binary gradient system was used for the rifamycin analysis with HPLC, with 0.5% Formic acid in H_2_O as mobile phase A and methanol as mobile phase B. For separation, the gradient was first kept at 95% A for 5 min, and then gradually decreased to 20% A in the next 10 min. After that, the gradient was held for 5 min at 20% A before being further increased to 95% A during the next 5 min. The total running time was 25 min and the flow rate was 0.3 mL/min. Rifamycin was detected at the wavelengths of 280 and 425 nm.

### Production of heterologously expressed recombinant RifQ

The *rifQ* coding sequence was amplified with RifQ-E1/RifQ-E2, using S699 genome DNA as the template, and the amplicon was digested with *Nde*I and *Xho*I, followed by being cloned into the same sites in pET22b vector, generating pET22b-RifQ for heterologous expression of RifQ. After being confirmed by Sanger sequencing, pET22b-RifQ was transformed into *Escherichia coli* BL21(DE3). Single clone was first grown in liquid LB medium overnight, and the cells were then inoculated (1% v/v) into to 500 mL fresh liquid LB. To induce the expression of RifQ, IPTG was added at a final concentration of 0.5 mM when OD_600_ reached 0.6, and cells were then cultured for another 6 h at 30 °C before being harvested by centrifugation at 5000 rpm for 10 min at 4 °C. His-tagged RifQ protein was purified by Ni–NTA gravity column and the homogeneity of the purified protein was confirmed by SDS-PAGE. The RifQ concentration was determined with the Bradford method [32].

### EMSA and DNase I Footprinting assay

The *rifP* promoter region was amplified from S699 genome DNA and cloned into pUC18B-T vector to obtain the template for preparation of the 5(6)-carboxyfluorescein (FAM) labeled probe, using primers of 5′-FAM labeled M13F(-47) and M13R. Probe was purified by the Gel and PCR Clean-Up System (Promega) and was quantified with NanoDrop 2000C (Thermo Fisher Scientific).

EMSA was performed according to the method described by Wang et al. [33], and 8 nM probe and different concentration of protein were added into a 20-µL reaction system. After incubation for 30 min at 37 °C, the reaction mix was loaded into the 5% native polyacrylamide gel buffered with 0.5× TBE. After electrophoresis, the gel was scanned with ImageQuant LAS 4000 mini (GE Healthcare Life Sciences). Sheared salmon sperm DNA was also added into each reaction system at a final concentration of 100 ng/µL to prevent non-specific binding between RifQ and DNA probes.

DNase I Footprinting assays were performed in the same procedure as we published before [33] and were carried out by Tolo Biotech. Specifically, 40 nM FAM-labeled probes were incubated with different amounts of protein in a total volume of 40 µL. Notably, 100 ng/µL sheared salmon sperm DNA was added in reaction system to prevent non-specific binding. Besides, when stated, other reagents such as rifamycin B, rifamycin SV and dimethyl sulphoxide (DMSO) were also added in the reaction system and the concentration of each reagent could be found in the labeling of the figures. After incubation at 37 °C for 30 min, 10-µL digest solution containing 100 nM freshly prepared CaCl_2_ and 0.015 unit DNase I (Promega) was added into the reaction system and the system was transferred to 25 °C and further incubated for 1 min. The reaction was then stopped by adding DNase I stop solution, and the samples were purified by the Gel and PCR Clean-Up System (Promega). The preparation of the DNA ladder, electrophoresis and data analysis were the same as previously described [33].

### Rifamycin feeding experiment

Strain *LYZL11* was a *rifA* frameshift mutant and produced no rifamycin. To perform the rifamycin feeding experiment, strain *LYZL11* was grown in liquid bennet and cultured at 30 °C till the stationary phase. After that, cells were inoculated into a fresh liquid bennet (5%, v/v) and cultured at 30 °C for 24 h before being supplemented with rifamycin B or SV to a final concentration of 2.5 mM. Cells were harvested at 12 and 24 h after the addition of rifamycins. Total RNA extraction and RT-PCR was performed as described as above.

### Primer extension analysis

TIS of *rifP* was determined by the primer extension assay, which was performed by Tolo Biotech. The culture of strain S699 and extraction of total RNA were the same as we described above. The *rifP* TIS was determined with both primers rifP-pe1 and rifP-pe2, which were complementary to the 29th to 50th and the − 399th to − 376th nucleotides (nts) relative to the *rifP* CDS, respectively. All primers were labeled with 5′-FAM and the RT products were analyzed with ABI 3130xl in the same protocol as described in the DNase I Footprinting assay [33]. The promoter region of *rifP* were cloned into the T-vector of pUC18B-T (Tolo Biotech.) [16] with primers of rifP-pF/rifP-pR, and the obtained plasmid (Additional file 5: Figure S5) was used as the template for sequencing analyses with FAM-labeled primers, following the protocol we described before [33].

### Determination of the protein molecular weight (MW) by gel filtration

RifQ MW was measured by size exclusion chromatography (SEC) using the Gel Filtration Calibration Kits (Low Molecular Weight, GE Healthcare Life Sciences). Blue Dextran 2000 was used to determine the void fraction, and Aprotinin (6.5 KD), Ribonuclease A (14 KD) and Ovalbumin (44 KD) were used as the MW markers. RifQ and the MW markers were run on SEC column Superdex 75 and the MW of RifQ was calculated according to the retention time.

## Additional files

**Additional file 1: Figure S1.** Sequence alignment analysis of RifQ and its homologue *Streptomyces virginiae* VarR (BAB32408.1).

**Additional file 2: Figure S2.** Transcriptional analysis of *rifQ* in *rifQ*+ and S699. The assay was performed at three different time points, i.e., 24 h, 48 h and 72 h. *rpoB* was used as an internal control.

**Additional file 3: Figure S3.** Diagram of the rifamycin biosynthesis cluster. The *rif* cluster consisted of 10 operons, which were indicated by solid lines, and direction of the operons was indicated by arrows.

**Additional file 4: Figure S4.** Transcriptional analysis of genes in *rif* cluster in different strains. *S699*, the wild type; *ΔrifQ*, the *rifQ* null mutant. At least one gene was chosen from each operon to characterize the transcriptional profile of the operon (*ref to* Figure S4), and *rpoB* gene was used as the internal control. All the transcription analyses were performed at both the early- and middle-exponential phases. Cycles for PCR amplification of each gene were labelled. Except *rifP*, which transcription increased in *ΔrifQ*, the transcription of all other tested genes showed moderate decrease at the early-exponential phase, indicating that RifQ might be involved in positive regulation of the transcription of these genes (or operons). However, more experiments may need to be done in future to uncover the exact role of RifQ in regulation of *rif* cluster. In addition, this moderate regulation mediated by RifQ disappeared when strains reached the middle-exponential phase.

**Additional file 5: Figure S5.** Primer extension analysis of *rifP* TIS. Primer rifP-pe1 was used and around 500-nt reverse transcript was observed via the ABI 3130xl capillary electrophoresis. Only one single peak was observed, demonstrating the high integrity of the *rifP* transcript. Based on this information, rifP-pe2 primer was further designed and the primer extension results employing rifP-pe2 could be found in Fig. 2b.

**Additional file 6: Figure S6.** Purification of recombinant His-tagged RifQ protein and measurement of its molecular weight (MW). (a) Purification of His-tagged RifQ via the Ni–NTA column, following the manufacturer’s instructions. Samples collected at different steps were analyzed by SDS-PAGE and stained by coomassie blue, employing the protein marker (Thermo Fisher Scientific; Cat. No. 26616). (b)  Diagram of the size exclusion chromatography analysis of RifQ, using the Low Molecular Weight (LMW) Kit (GE Healthcare Life Sciences).

**Additional file 7: Figure S7.** Schematic diagram of the *rifP* promoter region. TIS was obtained by primer extension assays (*ref to* Fig. 2), and was indicated by a curved arrow. The RifQ-protected region was underlined and the proposed RifQ-binding sites were boxed. The translation start codon of *rifQ* was shown in bold.

**Additional file 8: Figure S8.** Effect of rifamycins to the transcription of *rifP*. Rifamycin SV and rifamycin B were added into the *LYZL11* culture medium, respectively, and the *rifP* transcriptional level was measured at 24 h after the addition of rifamycins. DMSO was used as a blank control and *rpoB* was used as an internal control.

**Additional file 9: Table S1.** Strains and plasmids used in this study.

**Additional file 10: Table S2.** Primers used in this study.
