# Supplementary material for: A feedback regulatory model for RifQ-mediated repression of rifamycin export in Amycolatopsis mediterranei
Source: Microb Cell Fact. 2018 Jan 29;17:14. doi: 10.1186/s12934-018-0863-5 (PMC5787919; doi:10.1186/s12934-018-0863-5)
Supplement: Supplementary file 1 — Additional file 1: Figure S1. Sequence alignment analysis of RifQ and its homologue Streptomyces virginiae VarR (BAB32408.1). [file 12934_2018_863_MOESM1_ESM.docx]

**Figure S1. Sequence alignment analysis of RifQ and its homologue *Streptomyces virginiae* VarR (BAB32408.1).**
