# Supplementary material for: A feedback regulatory model for RifQ-mediated repression of rifamycin export in Amycolatopsis mediterranei
Source: Microb Cell Fact. 2018 Jan 29;17:14. doi: 10.1186/s12934-018-0863-5 (PMC5787919; doi:10.1186/s12934-018-0863-5)
Supplement: Supplementary file 2 — Additional file 2: Figure S2. Transcriptional analysis of rifQ in rifQ+ and S699. The assay was performed at three different time points, i.e., 24 h, 48 h and 72 h. rpoB was used as an internal control. [file 12934_2018_863_MOESM2_ESM.docx]

**
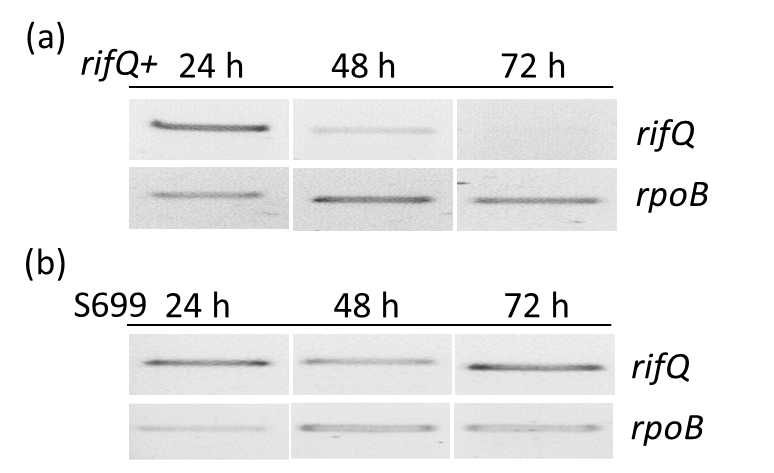
**

**Figure S2. Transcriptional analysis of *rifQ* in *rifQ+* and S699.** The assay was performed at three different time points, i.e., 24 h, 48 h and 72 h. *rpoB* was used as an internal control.
