# Supplementary material for: A feedback regulatory model for RifQ-mediated repression of rifamycin export in Amycolatopsis mediterranei
Source: Microb Cell Fact. 2018 Jan 29;17:14. doi: 10.1186/s12934-018-0863-5 (PMC5787919; doi:10.1186/s12934-018-0863-5)
Supplement: Supplementary file 3 — Additional file 3: Figure S3. Diagram of the rifamycin biosynthesis cluster. The rif cluster consisted of 10 operons, which were indicated by solid lines, and direction of the operons was indicated by arrows. [file 12934_2018_863_MOESM3_ESM.docx]

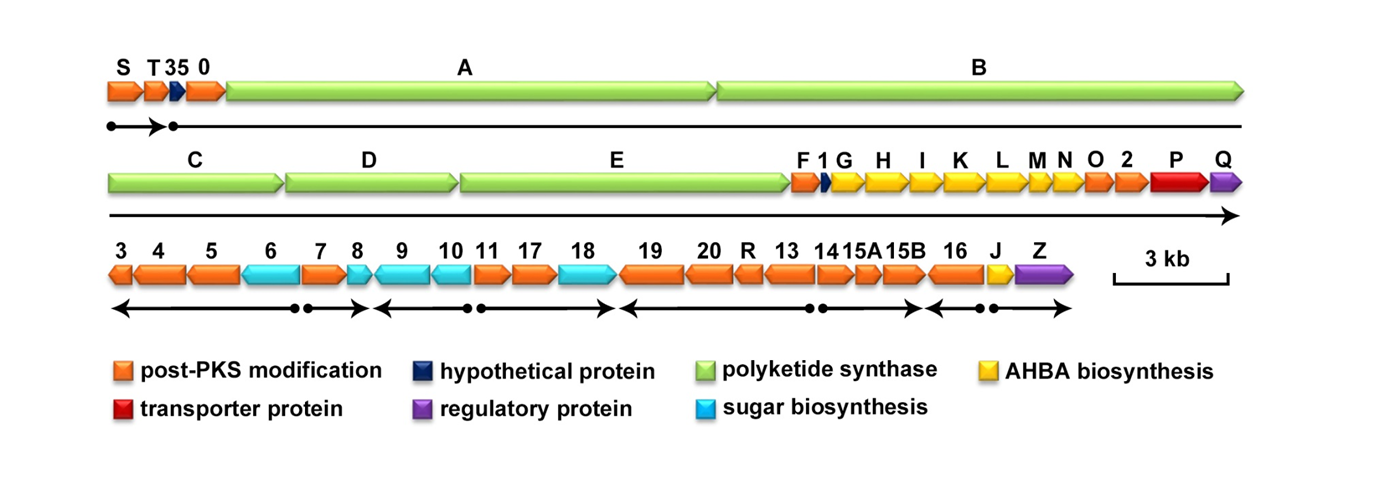


**Figure S3. Diagram of the rifamycin biosynthesis cluster.** The *rif* cluster consisted of 10 operons, which were indicated by solid lines, and direction of the operons was indicated by arrows.
