# Supplementary material for: A feedback regulatory model for RifQ-mediated repression of rifamycin export in Amycolatopsis mediterranei
Source: Microb Cell Fact. 2018 Jan 29;17:14. doi: 10.1186/s12934-018-0863-5 (PMC5787919; doi:10.1186/s12934-018-0863-5)
Supplement: Supplementary file 4 — Additional file 4: Figure S4. Transcriptional analysis of genes in rif cluster in different strains. S699, the wild type; ΔrifQ, the rifQ null mutant. At least one gene was chosen from each operon to characterize the transcriptional profile of the operon (ref to Figure S4), and rpoB gene was used as the internal control. All the transcription analyses were performed at both the early- and middle-exponential phases. Cycles for PCR amplification of each gene were labelled. Except rifP, which transcription increased in ΔrifQ, the transcription of all other tested genes showed moderate decrease at the early-exponential phase, indicating that RifQ might be involved in positive regulation of the transcription of these genes (or operons). However, more experiments may need to be done in future to uncover the exact role of RifQ in regulation of rif cluster. In addition, this moderate regulation mediated by RifQ disappeared when strains reached the middle-exponential phase. [file 12934_2018_863_MOESM4_ESM.docx]

**
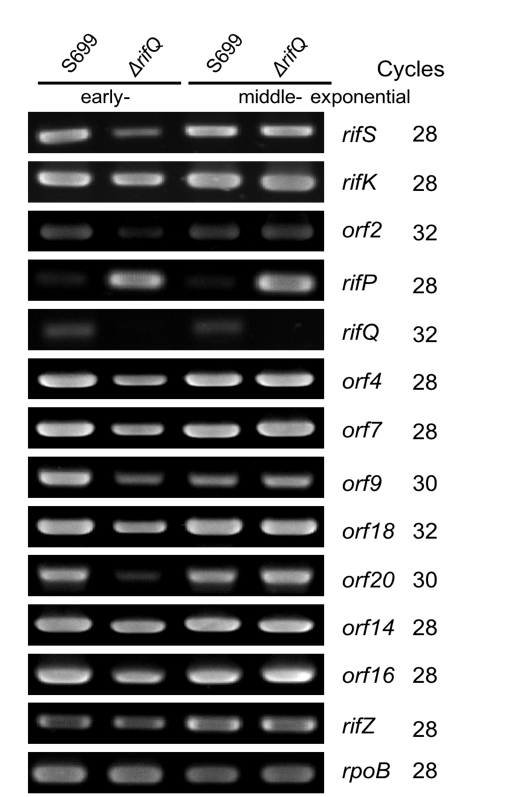
**

**Figure S4. Transcriptional analysis of genes in *rif* cluster in different strains.** *S699*, the wild type; *ΔrifQ*, the *rifQ* null mutant. At least one gene was chosen from each operon to characterize the transcriptional profile of the operon (*ref to* Figure S4), and *rpoB* gene was used as the internal control. All the transcription analyses were performed at both the early- and middle-exponential phases. Cycles for PCR amplification of each gene were labelled. Except *rifP*, which transcription increased in *ΔrifQ*, the transcription of all other tested genes showed moderate decrease at the early-exponential phase, indicating that RifQ might be involved in positive regulation of the transcription of these genes (or operons). However, more experiments may need to be done in future to uncover the exact role of RifQ in regulation of *rif* cluster. In addition, this moderate regulation mediated by RifQ disappeared when strains reached the middle-exponential phase.
