# Supplementary material for: A feedback regulatory model for RifQ-mediated repression of rifamycin export in Amycolatopsis mediterranei
Source: Microb Cell Fact. 2018 Jan 29;17:14. doi: 10.1186/s12934-018-0863-5 (PMC5787919; doi:10.1186/s12934-018-0863-5)
Supplement: Supplementary file 5 — Additional file 5: Figure S5. Primer extension analysis of rifP TIS. Primer rifP-pe1 was used and around 500-nt reverse transcript was observed via the ABI 3130xl capillary electrophoresis. Only one single peak was observed, demonstrating the high integrity of the rifP transcript. Based on this information, rifP-pe2 primer was further designed and the primer extension results employing rifP-pe2 could be found in Fig. 2b. [file 12934_2018_863_MOESM5_ESM.docx]

**Figure S5. Primer extension analysis of *rifP* TIS.** Primer rifP-pe1 was used and around 500-nt reverse transcript was observed via the ABI 3130xl capillary electrophoresis. Only one single peak was observed, demonstrating the high integrity of the *rifP* transcript. Based on this information, rifP-pe2 primer was further designed and the primer extension results employing rifP-pe2 could be found in Fig. 2b.
