# Supplementary material for: A feedback regulatory model for RifQ-mediated repression of rifamycin export in Amycolatopsis mediterranei
Source: Microb Cell Fact. 2018 Jan 29;17:14. doi: 10.1186/s12934-018-0863-5 (PMC5787919; doi:10.1186/s12934-018-0863-5)
Supplement: Supplementary file 6 — Additional file 6: Figure S6. Purification of recombinant His-tagged RifQ protein and measurement of its molecular weight (MW). (a) Purification of His-tagged RifQ via the Ni–NTA column, following the manufacturer’s instructions. Samples collected at different steps were analyzed by SDS-PAGE and stained by coomassie blue, employing the protein marker (Thermo Fisher Scientific; Cat. No. 26616). (b) Diagram of the size exclusion chromatography analysis of RifQ, using the Low Molecular Weight (LMW) Kit (GE Healthcare Life Sciences). [file 12934_2018_863_MOESM6_ESM.docx]

**Figure S6. Purification of recombinant His-tagged RifQ protein and measurement of its molecular weight (MW).** (a) Purification of His-tagged RifQ via the Ni-NTA column, following the manufacturer’s instructions. Samples collected at different steps were analyzed by SDS-PAGE and stained by coomassie blue, employing the protein marker (Thermo Fisher Scientific; Cat. No. 26616). (b)  Diagram of the size exclusion chromatography analysis of RifQ, using the Low Molecular Weight (LMW) Kit (GE Healthcare Life Sciences).
