# Supplementary material for: A feedback regulatory model for RifQ-mediated repression of rifamycin export in Amycolatopsis mediterranei
Source: Microb Cell Fact. 2018 Jan 29;17:14. doi: 10.1186/s12934-018-0863-5 (PMC5787919; doi:10.1186/s12934-018-0863-5)
Supplement: Supplementary file 7 — Additional file 7: Figure S7. Schematic diagram of the rifP promoter region. TIS was obtained by primer extension assays (ref to Fig. 2), and was indicated by a curved arrow. The RifQ-protected region was underlined and the proposed RifQ-binding sites were boxed. The translation start codon of rifQ was shown in bold. [file 12934_2018_863_MOESM7_ESM.docx]

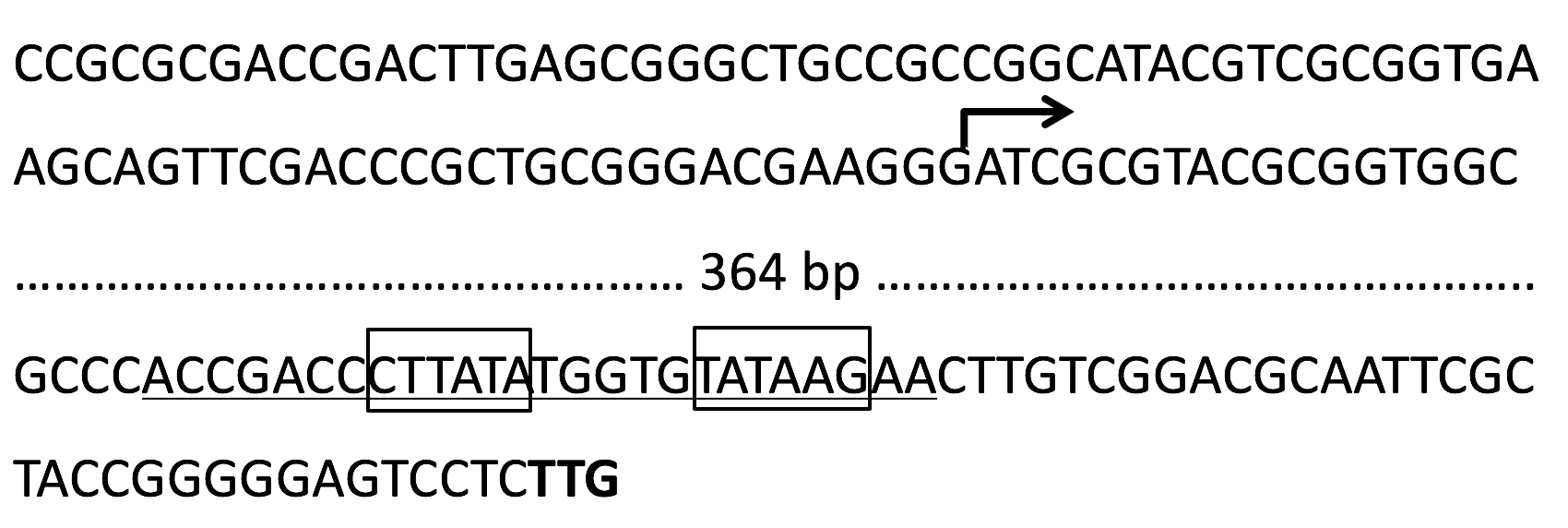


**Figure S4. Schematic diagram of the *rifP* promoter region.** TIS was obtained by primer extension assays (*ref to* Fig. 2), and was indicated by a curved arrow. The RifQ-protected region was underlined and the proposed RifQ-binding sites were boxed. The translation start codon of *rifQ* was shown in bold.
