# Supplementary material for: A feedback regulatory model for RifQ-mediated repression of rifamycin export in Amycolatopsis mediterranei
Source: Microb Cell Fact. 2018 Jan 29;17:14. doi: 10.1186/s12934-018-0863-5 (PMC5787919; doi:10.1186/s12934-018-0863-5)
Supplement: Supplementary file 8 — Additional file 8: Figure S8. Effect of rifamycins to the transcription of rifP. Rifamycin SV and rifamycin B were added into the LYZL11 culture medium, respectively, and the rifP transcriptional level was measured at 24 h after the addition of rifamycins. DMSO was used as a blank control and rpoB was used as an internal control. [file 12934_2018_863_MOESM8_ESM.docx]

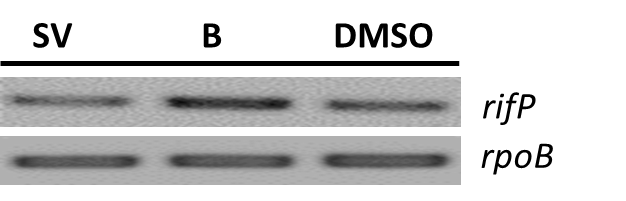


**Figure S8. Effect of rifamycins to the transcription of *rifP.*** Rifamycin SV and rifamycin B were added into the *LYZL11* culture medium, respectively, and the *rifP* transcriptional level was measured at 24 h after the addition of rifamycins. DMSO was used as a blank control and *rpoB* was used as an internal control.
