# Supplementary material for: A feedback regulatory model for RifQ-mediated repression of rifamycin export in Amycolatopsis mediterranei
Source: Microb Cell Fact. 2018 Jan 29;17:14. doi: 10.1186/s12934-018-0863-5 (PMC5787919; doi:10.1186/s12934-018-0863-5)
Supplement: Supplementary file 9 — Additional file 9: Table S1. Strains and plasmids used in this study. [file 12934_2018_863_MOESM9_ESM.docx]

**Table S1. Strains and plasmids used in this study.**

| Strains and plasmids | Description | Sources |
| --- | --- | --- |
| *E. coli* strains |  |  |
| DH5α | F-, φ80d*lacZ*ΔM15, Δ(*lacZYA*-*argF*)U169, *deoR*, *recA1*, *endA1*, *hsdR17* (rk-, mk+), *phoA*, *supE44*, λ-, *thi-1*, *gyrA96*, *relA1* | Invitrogen |
| BL21(DE3) | F-, *ompT*, *hsdS* (rB-, mB-), *gal*, *dcm* (DE3) | Invitrogen |
| *A. mediterranei* strains |  |  |
| S699 | The wild-type strain for production of rifamycin B | Our lab |
| *ΔrifQ* | S699 *rifQ* null mutant | This study |
| *rifQ+* | *ΔrifQ* complemented with pDXM-*rifQ* plasmid | This study |
| *vector+* | *ΔrifQ* complemented with pDXM4 blank plasmid | This study |
| *LYZL11* | A rifamycin-nonproducing strain, derived from partial deletion of *rifA* gene in *A. mediterranei* U32 | Our lab |
| plasmids |  |  |
| pET22b-RifQ | *rifQ* gene cloned into pET22b, which was used for heterologous expression of His-tagged RifQ | This study |
| pBC-Am | ColE1 ori, containing both chloramphenicol and apramycin resistance cassettes | Our lab |
| pUC18B-T | T vector derived from pUC18 plasmid | Tolo Biotech. |
| pUC18BT-*rifP*-P | The *rifP* promoter region cloned into pUC18B-T | This study |
| pUC18BT-*rifQ*-P | The *rifQ* promoter region cloned into pUC18B-T | This study |
| pUC18BT-*rifP*-PE | The *rifP* promoter region cloned into pUC18B-T, which was used for DNA sequencing analysis in the primer extension assay | This study |
| p*rifQko* | The *rifQ* knock-out cassette was cloned into pBluescript SK II vector, which was used for the construction of *rifQ* null mutant | This study |
| pDXM4 | An *A. mediterranei* replicating vector, with both apramycin and erythromycin resistance | Our lab |
| pDXM4-*rifQ* | pDXM4 vector containing the *rifQ* complementary cassette | This study |
