# Supplementary material for: A feedback regulatory model for RifQ-mediated repression of rifamycin export in Amycolatopsis mediterranei
Source: Microb Cell Fact. 2018 Jan 29;17:14. doi: 10.1186/s12934-018-0863-5 (PMC5787919; doi:10.1186/s12934-018-0863-5)
Supplement: Supplementary file 10 — Additional file 10: Table S2. Primers used in this study. [file 12934_2018_863_MOESM10_ESM.docx]

**Table S2. Primers used in this study**

| Names | | Sequences (5’-3’) |
| --- | --- | --- |
| For construction of RifQ expression plasmid | | |
| RifQ-E1 | | GGAATTCCATATGATGGGCAAACGGGCCTCGCAG |
| RifQ-E2 | | CACAAGCTTCCCGCCGGCGACCATGCCGGT |
| Cloning of promoters for preparation of FAM-labeled probes for EMSA and DNase I footprinting | | |
| rifPp1 | | CGTCGAGCTGCATCTGTTCCC |
| rifPp2 | | GCGAGATGACGGCCAGAATCAG |
| rifQp1 | | CGCCGTGCTCAACTCGCGGTTC |
| rifQp2 | | CCGTTTGCCCATCGTGGCCCTC |
| M13F(-47)FAM | | 5'-FAM-CGCCAGGGTTTTCCCAGTCACGAC-3' |
| M13R | | CAGGAAACAGCTATGACC |
| RT-PCR | |  |
| rifP-RTF | | CCGCACCCCATGCTCGAC |
| rifP-RTR | | CCAGCATTCCGCCGAACCC |
| RifQ-RTF | | GAGAGCGTGTGGCTTTCCCCG |
| RifQ-RTR | | TTCAGGTACTCGCCGTAGCAGC |
| rpoB-RTF | | ACGTCCAGATCCAGTCGTT |
| rpoB-RTR | CGTCCTTGTCGCTCGTCT |  |
| rifS-RTF | GGTTCTCCTTCTCCGCCTAC |  |
| rifS-RTR | CTCCCCTCACCGGCAGTTC |  |
| rifK-RTF | CCGGGCACCGAGGTCATC |  |
| rifK-RTR | TTGAGCCGCATGTTCGAGC |  |
| orf2-RTF | GGGGATCGCGGTGGTGTCGG |  |
| orf2-RTR | CGTCCCAGCTGATCTCGGCCT |  |
| orf4-RTF | TCCGGTGGACCTGCTCAAGA |  |
| orf4-RTR | ACGTCGGGCGGTGCCAATC |  |
| orf7-RTF | GTGCTGGAACCGTGGATGTA |  |
| orf7-RTR | AGGACGCTTCCCGGACGAG |  |
| orf9-RTF | ACGCAACGGAATCCGCCATC |  |
| orf9-RTR | GCCGACATCGTGCCCATCAG |  |
| orf18-RTF | GTGCTCGGCATCCTCGTCAA |  |
| orf18-RTR | GCGTGCCCGCGTCACTGCG |  |
| orf20-RTF | TGGCGATCCAGCACAGTGTC |  |
| orf20-RTR | GCTTCGGGTCCCACGGTTT |  |
| orf14-RTF | ACCGCGAACGACGTCATGGT |  |
| orf14-RTR | GATGTGCTGCAGGGCCTGTT |  |
| orf16-RTF | GTTCATGCAGGCGCTGGTCA |  |
| orf16-RTR | GCGAGTGCACCCCGTAAGTC |  |
| rifZ-RTF | GCAGCCTGCTGTTCGCCA |  |
| rifZ-RTR | GCGCTCACCGACGTGTACG |  |
| Primer extension | |  |
| rifP-PF | | TACTACGCAGCCCCGGCCCGC |
| rifP-PR | | GAAACGTGCCCGGGAACAGATGC |
| rifP-PE1 | | CGTGGATTCCGGGTTTCGGTC |
| rifP-PE2 | | GAAACGTGCCCGGGAACAGATGC |
| rifQ-PE | | GACGATCTTTTCGCGGTCGAGC |
| *rifQ* knock out and complementation | | |
| rifQkoP1 | | GGCTGCAGGAATTCGATTGGTCACCTCCGGCGTCGTC |
| rifQkoP2 | | CTGCAAATACGGCATCACGGGGAAAGCCACACGCTCTCG |
| rifQkoP3 | | TGATGCCGTATTTGCAGTACCAG |
| rifQkoP4 | | TTTACCCGCAGGACATATCCAC |
| rifQkoP5 | | ATGTCCTGCGGGTAAATGGCTGTTCACCGGCATGGTCGC |
| rifQkoP6 | | GTATCGATAAGCTTGATCGCCCGATCGCCGAGGTCAC |
| rifQkoCK1 | | CGCCGTGCTCAACTCGCGGTT |
| rifQkoCK2 | | CTCACCGACCGCGAAGGGAC |
| rifQkoCP1 | | TGCTGTCGGAATGGACGATTCAGCCGGCGTGAGCAGGAGG |
| rifQkoCP2 | | CTGCGAGGCCCGTTTGCCCAAGAGGACTCCCCCGGTAGC |
| rifQkoCP3 | | GGCAAACGGGCCTCGCAGC |
| rifQkoCP4 | | AGAAGTGATGCACTTTGATCGCAGCGTCGACTACGGCAAG |
